# Development of 3D MRI-Based Anatomically Realistic Models of Breast Tissues and Tumours for Microwave Imaging Diagnosis

**DOI:** 10.3390/s21248265

**Published:** 2021-12-10

**Authors:** Ana Catarina Pelicano, Maria C. T. Gonçalves, Daniela M. Godinho, Tiago Castela, M. Lurdes Orvalho, Nuno A. M. Araújo, Emily Porter, Raquel C. Conceição

**Affiliations:** 1 Instituto de Biofísica e Engenharia Biomédica, Faculdade de Ciências, Universidade de Lisboa, Campo Grande, 1749-016 Lisbon, Portugal; dmgodinho@fc.ul.pt (D.M.G.); rcconceicao@fc.ul.pt (R.C.C.); 2 Departamento de Radiologia, Hospital da Luz Lisboa, Luz Saúde, 1500-650 Lisbon, Portugal; tacastela@hospitaldaluz.pt (T.C.); lorvalho@hospitaldaluz.pt (M.L.O.); 3 Centro de Física Teórica e Computacional, Faculdade de Ciências, Universidade de Lisboa, Campo Grande, 1749-016 Lisbon, Portugal; nmaraujo@fc.ul.pt; 4 Department of Electrical and Computer Engineering, The University of Texas at Austin, 2501 Speedway, Austin, TX 78712, USA; emily.porter@austin.utexas.edu

**Keywords:** realistic numerical models, breast tumour models, dielectric properties, image segmentation, breast model repository for microwave diagnosis

## Abstract

Breast cancer diagnosis using radar-based medical MicroWave Imaging (MWI) has been studied in recent years. Realistic numerical and physical models of the breast are needed for simulation and experimental testing of MWI prototypes. We aim to provide the scientific community with an online repository of multiple accurate realistic breast tissue models derived from Magnetic Resonance Imaging (MRI), including benign and malignant tumours. Such models are suitable for 3D printing, leveraging experimental MWI testing. We propose a pre-processing pipeline, which includes image registration, bias field correction, data normalisation, background subtraction, and median filtering. We segmented the fat tissue with the region growing algorithm in fat-weighted Dixon images. Skin, fibroglandular tissue, and the chest wall boundary were segmented from water-weighted Dixon images. Then, we applied a 3D region growing and Hoshen-Kopelman algorithms for tumour segmentation. The developed semi-automatic segmentation procedure is suitable to segment tissues with a varying level of heterogeneity regarding voxel intensity. Two accurate breast models with benign and malignant tumours, with dielectric properties at 3, 6, and 9 GHz frequencies have been made available to the research community. These are suitable for microwave diagnosis, i.e., imaging and classification, and can be easily adapted to other imaging modalities.

## 1. Introduction

Female breast cancer was the most common cancer diagnosed worldwide in 2020, with over 2.26 million new cases [1]. Breast cancer was also reported the fifth deadliest type of cancer in 2020, and the cancer with the highest mortality rate in the female population [1]. Early detection and intervention have been identified as determining factors in the reduction of breast cancer mortality rates and are the key factors for a successful treatment outcome, thereby improving the quality of life of cancer patients and survival rates [2].

The most common imaging modality for breast cancer detection is X-ray mammography [2,3]. Although mammography is still the go-to imaging method for cancer screening, it does not provide reliable results for women with dense breasts, which are common among younger women [4]. Sensitivity of mammography exams range from 62.9%, for women with extremely dense breasts, to 87.0%, in women with mostly fatty breasts. Specificity has been reported to range from 89.1% to 96.9% for the same breast types [5]. Besides showing different sensitivities depending on tissue density, mammography also requires the use of ionizing radiation, and an uncomfortable breast compression.

Microwave Imaging (MWI) has been identified as a technique that can tackle the shortcomings of X-ray mammography [6,7]. In MWI, an external electromagnetic field is applied onto the region of interest in the body, resulting in electromagnetic scattering generated by tissues with different dielectric properties. The conductivity and relative permittivity are the most relevant dielectric properties of biological tissues, where the latter is intrinsically related to the water content present in the tissue sample. In recent years, MWI systems have been studied for early-stage breast cancer diagnosis [8,9,10] due to the contrast between the dielectric properties of cancerous and healthy breast tissues at microwave frequencies [11]. Cancerous tissues differ from healthy tissues due to permeability changes in tumour cell membrane causing an increase of water flow to the interior of the cell. Hence, the extra quantities of water and dissolved ions inside the cancerous cells lead to greater values of relative permittivity and conductivity when compared to healthy cells of the same tissue type [12,13,14,15]. In addition to being a comfortable and non-invasive imaging modality, MWI devices are also portable, low-cost, user-independent, use low-power, and therefore, are suitable for screening programmes.

Simulations and experiments mimicking the realistic conditions and realistic patients of a clinical exam are essential for the development and validation of MWI diagnostic systems, as well as the development of therapeutic devices, such as those for microwave hyperthermia and ablation [16,17]. Hence, realistic computational and physical models of breast tissues and tumours with the respective dielectric properties estimated at microwave frequencies are of utmost importance.

Realistic models of healthy breast tissues are currently available in public repositories, however, due to their complex shape, tumour models are often oversimplified. Malignant breast tumours generally have an irregular shape surrounded by spicules whereas benign tumours present roughly rounded or elliptical shapes [18,19]. Therefore, we aim to provide the scientific community with a repository of multiple anthropomorphic models of breast tissues with realistic benign and malignant breast tumours.

In this study, we use MRI exams to develop the anatomically realistic models of the breast, and estimate the dielectric properties of fat, fibroglandular, skin, muscle, and tumorous tissues of this region. Additionally, we developed a robust segmentation pipeline suitable to segment highly heterogeneous tumours from MRI exams. The developed semi-automatic image processing pipeline includes the following steps: (i) Image pre-processing, (ii) Image segmentation/Feature extraction, comprising the breast region (skin, breast/chest wall boundary, fat and fibroglandular tissues) and the breast tumour, and finally, (iii) Estimation of tissue dielectric properties. The second aim of our work is to address the lack of realistic breast tumour phantoms for MWI prototype testing. Hence, we have been preparing accurate models of the breast which will be used to simulate tissue response to microwave radiation and later 3D printed for experimental testing. In short, our work aims to:

- provide the scientific community with a repository of multiple anthropomorphic models of breast tissues and tumours;
- address the lack of realistic physical breast tumour phantoms for MWI prototype testing.

This paper is organised as follows: firstly, we present related work already conducted concerning breast and breast tumour models; then, we detail the materials used and the methodology developed for image pre-processing, segmentation, and estimation of dielectric properties; then, we present the results of our proposed methodology, followed by a discussion, and finally, we highlight the main conclusions of our work.

### Related Work

Winters et al. [20] designed two numerical MRI-derived models of the breast surface. Later, a public repository with nine numerical breast phantoms containing realistic distributions of skin, fat, glandular and muscle tissue was created [21]; the STL files to 3D print these models was later published in [22]. More recently, an anthropomorphic breast model repository including realistic models of skin, fat and fibroglandular tissues, and a model of a 10 mm malignant tumour was made available in [23]. However, the segmentation of the tumour volume was achieved by manual cropping and thresholding techniques.

Studies including numerical breast tumour models often oversimplify their representation by using spherical [24,25,26], and cross- and peanut-shaped numerical models [14]. A more sophisticated 3D breast mass model was developed in [27] using a growth model with a dense centre and fading boundaries from an ellipsoid volume which resulted in a stellate pattern.

Most of the physical breast tumour models reported in the literature to test MWI systems present an unrealistic simplified shape, generally spherical, elliptical, and cylindrical: in [22], spherical glass bulbs with 5, 10 and 15 mm radii containing saline solutions were used to mimic tumour tissues in phantom studies; an ellipsoid container with internal dimensions 10 and 20 mm was 3D printed in [28] to study the feasibility of a radar-based breast MWI dry setup; a 20 mm spherical phantom filled with a 10:90 ratio of water to glycerine was used to emulate a breast tumour in [29]; two cylindrical shaped tumour phantoms with 10 and 20 mm diameters and 30 mm height were tested in [30]; and in [31], a small cylindrical plastic container filled with water was used as a breast tumour phantom in experimental tests.

Breast tumours have been modelled, more realistically, with Gaussian Random Spheres. These were used for validation tests of a microwave imaging device in [32], and for tumour classification using a MWI prototype system in [33]. In [34], realistic benign and malignant breast phantoms were carved by hand, resulting in approximate spherical and spiculated models for benign and malignant tumours, respectively. We emphasize that none of the models reported in the literature were based on accurate anatomical representations of tumours.

A summary of previously developed methodologies to create breast models has been recently published in [35].

## 2. Materials and Methods

### 2.1. Dataset

This study was approved by the Scientific and Ethical Commission of Hospital da Luz—Lisboa, under references CES/44/2019/ME (19 September 2019) and CES/34/2020/ME (6 November 2020). The breast MRI exams collected at Hospital da Luz—Lisboa were anonymised before processing and informed consent was obtained from all patients.

The current dataset of this on-going study comprises exams from 16 patients, with a total of 29 tumours: 15 benign and 14 malignant. In this paper, we selected breast masses scored with BI-RADS 2 and 3 [1] (benign tumours) and BI-RADS 5 and 6 (malignant tumours) [1].

Women were imaged in a prone position in a 3.0T MAGNETON Vida clinical Magnetic Resonance (MR) scan (Siemens Healthineers, Erlangen—Germany) with a dedicated coil for the breast (Siemens Breast 18 coil, Siemens Healthineers, Erlangen—Germany). Two MRI sequences were collected: Dynamic Contrast Enhanced (DCE) transversal three-dimensional (3D) T1-weighted (T1-w) Fast Low Angle Shot 3D (fl3D) Spectral Attenuated Inversion Recovery (SPAIR) sequence; and Direct coronal isotropic 3D T1-w fl3D Volumetric Interpolated Breath-hold Examination (VIBE) Dixon image sequence (T1-w Dixon).

DCE-fl3D consists of a fat-suppression sequence with six sets of images: a pre-contrast image, acquired before the injection of gadolinium intravenous contrast agent, and five post-contrast consecutive images where highly vascularised tissues, such as tumours, are enhanced. Digital subtractions of each post-contrast image from the pre-contrast image are also available. The digital subtractions enhance tumour regions due to the contrast uptake in those locations and annul hypersignal regions present in the pre-contrast image. These images present high resolution in all anatomical planes and have isotropic voxels. Even though our dataset includes images with different spatial resolutions (0.99 mm × 0.99 mm × 1 mm and 1.04 mm ×1.04mm × 1 mm), we only use images with 0.99 mm × 0.99 mm × 1 mm in this paper. We chose the subtraction DCE-fl3D image (SUB-DCE-fl3D) that best reveals the whole tumour region for tumour segmentation. One must note that larger tumours require more time delay for contrast enhancement to be observed.

The T1-w Dixon sequence relies on the difference in resonance frequency between hydrogen nuclei bound to water and fat. This difference allows obtaining four sets of images in a single acquisition: in-phase, opposite phase, fat-only, and water-only images. For this study, we used fat-only (F), water-only (W), and in-phase (I) images to retrieve structural information of the breast. To derive the dielectric properties of the tissues in the breast (fat, fibroglandular, skin, and benign and malignant tumours), we used the T1-w Dixon-I images, as the fat and fibroglandular tissues are easier to distinguish in their histograms. These images had isotropic voxels (0.99 mm × 0.99 mm × 1 mm) and were acquired in the coronal plane.

### 2.2. Pre-Processing Pipeline

This section details each step of the pre-processing pipeline applied to breast MRI images before tissue segmentation.

#### 2.2.1. Image Registration

We used two different MRI sequences to segment the breast tissues: the transverse SUB-DCE-fl3D sequence (for tumour segmentation) and the coronal T1-w Dixon sequence (for the segmentation of fat, fibroglandular, skin, and the breast/chest wall boundary, predominantly composed of muscle); hence the alignment of the two images is required. We used the Insight Toolkit (ITK) implementation (SimpleITK’s) [36] of a linear registration with linear interpolation to register the SUB-DCE-fl3D sequence (moving image) to the T1-w Dixon sequence (static image). T1-w Dixon was considered the static image due to its higher information content regarding the different breast tissues. The application of the linear transformation resulted in images with dimensions and resolution of the static image, and in the same spatial referential, allowing their correct superimposition.

#### 2.2.2. Bias Field Correction

MRI images are prone to the bias field artefact [37], which causes unreliable intensity variations within voxels of the same tissue. As the accuracy of intensity-based imaging processing algorithms, such as segmentation and classification, is greatly affected by the bias field artefact, a pre-processing step addressing its effects and correction is required [38].

A nonparametric nonuniform signal intensity normalisation (N3) algorithm, proposed by Sled et al. [39], uses a Gaussian model to correct the bias field without the need for a priori knowledge. Later, an improvement of this technique led to the development of the N4 algorithm [40], which uses a multi-scale optimisation approach to compute the bias field. This algorithm has shown promising results in removing the bias field from breast MRI images [41]. The bias field artefact correction was applied to all images using the SimpleITK N4BiasFieldCorrectionImageFilter implementation [40].

#### 2.2.3. Data Normalisation

Subsequently, the images were scaled between 0 and 255 using the Minimum-Maximum (Min-Max) normalisation approach, following Equation (1):

(1) $$v^{\prime }=\frac{v-\min\left(A\right)}{\max\left(A\right)-\min\left(A\right)}\left(new_{\max\left(A\right)}-new_{\min\left(A\right)}\right)+new_{\min\left(A\right)},$$

where 𝑣′ and 𝑣 are the original and scaled values of each voxel respectively; 𝐴 is the volume data, and $\max\left(A\right)$ and $\min\left(A\right)$ are the maximum and minimum values of 𝐴, respectively. $new_{\max\left(A\right)}$ and $new_{\min\left(A\right)}$ are the [new] maximum and minimum values of the scaled range, respectively [42].

#### 2.2.4. Image Filtering

MRI images are prone to noise due to image acquisition errors, which corrupts their quality and deteriorates the performance of intensity-based automatic segmentation algorithms. Salt-and-Pepper noise, commonly present in MRI images, consists of randomly distributed corrupted voxels which were either set to have the value 0 or the maximum intensity value of the voxels in the image [43]. The median filter is a well-known non-linear filter which allows the replacement of the value of a voxel by the median of the gray levels in its neighbourhood and has been proven very effective in the presence of Salt-and-Pepper noise [44]. Besides removing noise, the implemented 3-by-3-by-3 median filter, applied to the SUB-DCE-fl3D images, also smooths voxel signal intensity differences between tumorous and non-tumorous tissues. However, we did not apply the median filter for infra-centimetric tumours, as it produces substantial changes in the size and shape of tumours.

### 2.3. Image Segmentation

Accurate breast tissue segmentation is of paramount importance to obtain anatomically realistic numerical and physical breast models. Most tissue segmentation algorithms rely on the discontinuity or similarity properties of the image’s intensity values [44,45]. Several processing pipelines have been developed to identify the different tissues of the breast and use a combination of both properties to achieve a correct segmentation. Discontinuity-based approaches for breast tissue segmentation rely on the identification of air-breast and breast-chest wall boundaries [46,47], while similarity-based techniques mostly rely on thresholding [48], region-growing [47], and clustering [49,50]. Energy-based approaches, such as active contour, have also been proposed to segment the skin of the breast [51].

**Figure 1:**
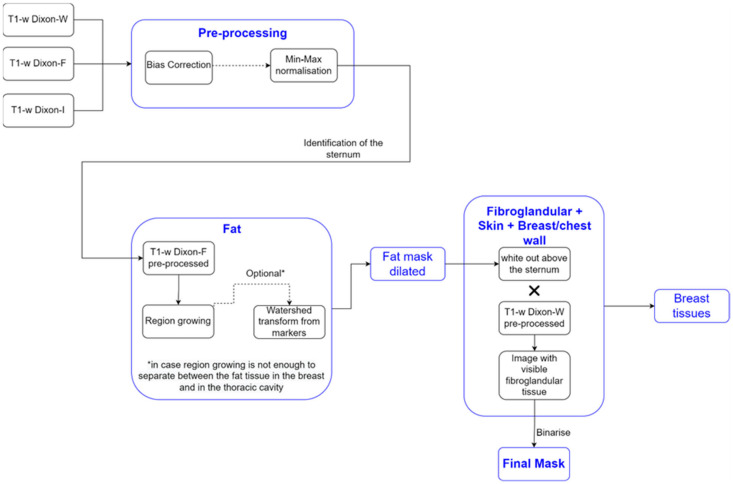
Simplified schematic of the processing steps to obtain the mask of the breast region.

We detail the segmentation methodology used for the different breast tissues in this section. Figure 1 represents a simplified schematic of the steps followed to obtain a mask of the breast region.

#### 2.3.1. Breast Region

We used T1-w Dixon-F and T1-w Dixon-W images to generate a binary mask of the breast region. We chose these images since fat is represented with high-intensity values in the T1-w Dixon-F images, while fibroglandular, skin, and muscle have high-intensity values in the T1-w Dixon-W images. We present the results from our processing pipeline in Section 3.2.1, for clarity.

1. Step 1: Fat mask + removal of organs inside the thoracic cavity

The organs inside the thoracic cavity, such as the heart, lungs, stomach, and liver, presented low-intensity values compared with the high-intensity values of the fat tissues in the T1-w Dixon-F image. Hence, we used these images to exclude them from the breast region mask.

Firstly, we needed to locate the sternum in the T1-w Dixon-W image. To do this, we identified, in each frame, the voxels with an intensity higher than the mean intensity of the T1-w Dixon-W image. We then picked the coordinates of the outer-most identified voxel and used these to later compute the seed to grow the fat region.

We applied a region growing algorithm, implemented using SimpleITK’s NeighborhoodConnectedImageFilter, to the T1-w Dixon-F image to identify the voxels connected to the seed and whose neighbours lie within a user-defined intensity range. We automatically calculated the intensity range assigned to fat tissue. The lower limit has been identified by other authors [47] as the sum of the mean and three times the standard deviation values of the voxel intensities of non-fat suppressed breast MRI images, where fat, fibroglandular, muscle, and skin tissues are represented with medium to high-intensity values. Since only fat has high-intensity values in the T1-w Dixon-F image, we adjusted the lower limit of the intensity to the sum of the mean and standard deviation values of the voxel intensities. We set the upper limit to the maximum intensity voxel of the image. In breast MRI images, with no clear separation between the fat tissues of the breast and the inner part of the thoracic cavity, the region growing algorithm may not correctly segment both fat tissues. However, the application of morphological watershed transform from markers [52] (the marker image obtained from the output of the region growing algorithm by applying a distance transform) to the region growing algorithm output successfully distinguishes the two types of fat.

2. Step 2: Skin + Fibroglandular + Fat mask

We included the skin by dilating the fat mask. According to [53], the thickest skin area of the breast corresponds to the areolar region with 2.04 ± 0.31 mm. With a voxel spacing, in millimetres, of 0.9965 × 0.9965 × 1, we performed a dilation using a structuring element of radius 3. The dilated mask was whited out on the anterior part of the body and then multiplied by the T1-w Dixon-W image to include the fibroglandular tissue. We binarized the resulting image by thresholding, using statistical information (*mean* and *standard deviation*) of the image, and added the binarized image to the dilated mask. The threshold was defined, in Equation (2), as:

(2) $$threshold=mean+\frac{standard\;deviation}{4}.$$

To binarize the image, we had to consider the following: the threshold had to be higher than the mean voxel intensity to remove the background voxels introduced by multiplication with the T1-w Dixon-W image, but not excessively high that the holes left from the fat mask were not filled, especially in the nipple area. From this step, we obtain a mask that includes the skin, fat, and fibroglandular tissue.

3. Step 3: Mask evaluation

As our methodology uses the sternum coordinates as reference, areas where a tumour invades the pectoralis muscle are not included in the breast region mask. An invasive tumour introduces an asymmetry in the breast; hence, the obtained breast mask and its flipped image (flipped along the sagittal plane) are evaluated. We chose the mean-squared error (*MSE*) metric (Equation (3)) to evaluate the differences between the mask and its flipped image:

(3) $$MSE=\left(\frac{1}{n}\sum_{i=1}^{n}{\left(A_{i}-B_{i}\right)}^{2}\right)\times 100,$$

where n represents the number of data points, *i* varies from 1 to *n*, A is the mask, and B is the flipped image of the mask.

The lower the mean-squared error, the more similar are the images under evaluation. We considered that a mean-squared error value higher than 10% indicated the presence of an invasive tumour.

4. Step 4: Mask for an exam with an invasive tumour (optional, when *MSE* > 10%)

For a mean-squared error value higher than 10%, we added the breast region mask and its flipped image. Then, we compared non-coinciding areas to the T1-w Dixon-I image and reassigned voxels with intensity lower than the mean voxel intensity of the T1-w Dixon-I image to 0. After this process, the breast mask becomes symmetric in the breast/chest wall boundary region (mask for invasive tumours). To close any holes left from the process of reassigning the voxels to 0, we applied SimpleITK’s BinaryFillholeImageFilter to the symmetric breast mask.

5. Step 5: Segmentation of skin + breast/chest wall boundary

We used the breast mask contour to identify the skin and the breast/chest wall boundary. We scanned it to retrieve the first white voxel from left to right, right to left, and top to bottom in the sagittal plane. The resulting image corresponded to a 1-voxel-thick skin contour, where some areas in the sagittal centre of the body, between the breasts, were not included. A 1-voxel-thick breast/chest wall boundary contour corresponded to the largest-connected component resulting from the subtraction between the breast mask contour and the skin contour, followed by blacking out the voxels in the anterior part of the body, above the sternum.

The final skin contour corresponded to the largest connected component of the subtraction between breast mask contour and the 1-voxel-thick breast/chest wall boundary contour. We obtained the final breast/chest wall boundary contour by subtracting the final skin contour from the breast mask contour.

As mentioned in Section 2.3.1, we dilated the fat mask obtained from applying region growing to the T1-w Dixon-F image, using a structuring element of radius 3. As a result, it is necessary to dilate the contours by the same structuring element to estimate the skin and breast/chest wall boundary tissues. After the dilation, we multiplied the resulting image by the original mask to remove the “excessive” breast area.

For an invasive tumour exam, we adjusted the method to identify the breast/chest wall boundary tissues by building upon the breast region mask that was made symmetric in the sagittal plane. We obtained the breast/chest wall boundary tissues by subtracting the mask for invasive tumours and the “original” mask (the mask obtained before its addition with its flipped image, as obtained in Step 4), followed by dilation with a structuring element of radius 3. Although the breast/chest wall boundary is predominantly muscle, it may include other nearby tissues.

6. Step 6: Skin evaluation

Since the skin has varying thickness throughout the breast—ranging from 2.35 mm in the areolar region to 1.52 mm in the lateral quadrant [53]—we needed to adjust the skin mask to portray an accurate skin thickness. Firstly, we multiplied the T1-w Dixon-W image by the negated fat mask to obtain an image with skin and fibroglandular tissue. Then, we binarised the resulting image by thresholding, and multiplied the binarised image by the dilated skin contour.

7. Step 7: Fibroglandular tissue segmentation

With the fat tissue, skin, and breast/chest wall boundary separately segmented, the fibroglandular tissue can be identified by subtracting those tissues from the whole breast mask. In this work, we further applied the Gaussian Mixture Model described in [53] to segment both fat and fibroglandular tissues into sub-categories (low, median, and high), allowing to incorporate tissue heterogeneity, as reported in [54].

#### 2.3.2. Tumour Segmentation

Accurate tumour segmentation is of utmost importance for tumour evaluation and extraction of its characteristics. This task is very challenging as breast lesions widely vary in shape and intensity distribution. Strategies based on data clustering, particularly unsupervised clustering methods such as K-means and Fuzzy C-means, have been used for breast tumour segmentation using MRI exams [55,56,57,58]. Such methods group a set of data objects of the whole image/volume into clusters by maximizing intraclass similarity and minimizing interclass similarity. The proposed approaches using K-means in [55,56] and Fuzzy C-means in [57,58] outperformed standard techniques and showed high accuracy in segmenting breast tumours. A semi-automatic algorithm using a marker-controlled watershed method proposed in [59] was proven more efficient in connecting disjoint areas in lesions compared to classical K-means clustering and Gaussian Mixture Model clustering [60]. Thakran et al. [61] developed an automatic methodology for breast tumour segmentation based on Otsu thresholding.

All previous methodologies rely on intensity values of the whole image to obtain the region of interest (tumour), as they were developed under the assumption that, generally, biological tissues are fairly well separated within a grayscale image. However, in the case of heterogeneous structures comprising a wide range of intensity values, such as heterogeneous malignant tumours, these methods cannot include all voxels comprising the tumour within the same cluster, leading to poor segmentation of the tumour volume.

In this work, we used a 3D region growing algorithm, based on [62], to address tumours with voxels spreading over a wide range of intensity values. As previously mentioned in Step 1, this method uses a seed to grow the region to adjacent points based on an intensity threshold criterion. Such criterion defines the intensity range of the voxels in the growing region as the seed intensity value ± threshold.

In SUB-DCE-fl3D images, tumour regions generally present higher intensities when compared to neighbouring tissues, so we used the highest intensity voxel as the seed in the region growing algorithm. For that, one must select a slice (per tumour) in which the algorithm should choose the seed voxel. Although, most of the time, the highest intensity voxel corresponds to the tumour, it is possible that in some cases this will not be true—a manual assignment may be required. For the choice of the threshold value, one should confirm that the seed value minus the threshold corresponds to the minimum intensity value of the tumour voxels; hence only voxels belonging to the tumour will be included in the growing region. Experiments using our dataset showed that a lower bound of threshold defined by the mean minus three times the standard deviation of all body voxels provided good results.

**Figure 2:**
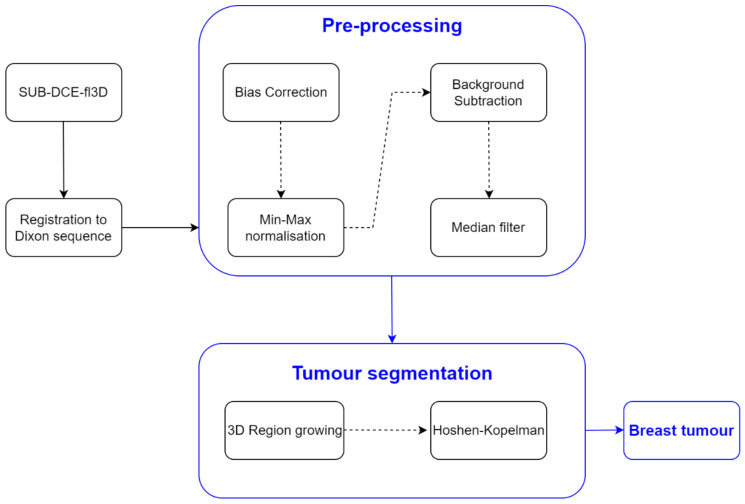
Simplified schematics of the processing steps to obtain the mask of the breast tumours.

Oftentimes, the 3D region growing algorithm was unable to segment the tumour exclusively, especially for heterogenous tumours with a wide range of voxels’ intensities. Instead, other body tissues were included in the growing region. Hence, in order to segment only the tumour, the Hoshen-Kopelman algorithm was applied [63] to retrieve the largest connected component. A schematic of the methodology used for breast tumour segmentation is shown in Figure 2.

### 2.4. Dielectric Properties Estimation

We performed the dielectric properties estimation by assigning the voxel intensities of the different tissues of the breast (fat, fibroglandular, skin, muscle, and tumour), in the T1-w Dixon-I image, to the corresponding dielectric properties reported in the literature via piecewise-linear mapping [21,64,65].

Relative permittivity and conductivity curves of single-pole Debye model have been described [21,64,65] for each tissue type of the breast (fat, fibroglandular, tumour, skin, and muscle). In addition, we further separated the fat and fibroglandular tissues into six categories with the corresponding dielectric property curve: fibroglandular-low, fibroglandular-medium, fibroglandular-high, fat-low, fat-medium, and fat-high. To obtain an appropriate mapping between the voxel intensities and the corresponding dielectric properties, we fitted a two-component Gaussian Mixture Model to the histogram of the pre-processed images, adapted from [21]. The histogram contains the contribution of fat and fibroglandular tissues as defined in Equation (4), for fat, and Equation (5), for fibroglandular tissue, by the Gaussian Mixture Model method,

(4) $$\gamma_{fat}=\left(\mu_{fat},\delta_{fat}{}^{2}\right)$$

(5) $$\gamma_{fg}=\left(\mu_{fg},\delta_{fg}{}^{2}\right),$$

where $\mu_{x}$ and $\delta_{x}{}^{2}$ represent the mean and variance of each distribution. The remaining parameters are defined in Table 1. Skin also has specific dielectric curves reported in the literature [64,65].

When a histogram does not show sufficient separation between the Gaussian curves corresponding to the contributions of the fat and fibroglandular tissues, we used the parameters in Table 2, where *δ* is a user-defined positive scalar [21]. In our work, we define δ as in Equation (6),

(6) $$\delta =\frac{Fat\_low-Fibroglandular\_high}{2}$$

For each segmented tissue type, the minimum and the maximum value of the intensity voxels of the MRI exam are associated with the lower- and upper-bound curve of each tissue, respectively. At each frequency, the remaining voxels are linearly mapped to a value between the curves of that tissue using a piecewise linear interpolation.

Table 3 contains single-pole Debye parameters (permittivity at high frequency—$\varepsilon_{\infty }$, the difference between static permittivity and permittivity at high frequency—Δε, relaxation time—τ, and static conductivity—$\sigma_{s}$) of the dielectric curves for each tissue type [64,65]. As reported in [64,65], Debye parameters are only valid for frequencies between 3 and 10 GHz. We assumed that the breast/chest wall boundary was composed of muscle.

According to Lazebnik et al. [15], the dielectric properties of benign tissues are similar to the properties of lower-adipose-content normal breast tissues, hence, we used the same linear interpolation obtained from the whole image to estimate the dielectric properties of benign tissues. Regarding malignant breast tumours, the 1-pole Cole-Cole parameters of the dielectric property curves are reported [15]. Two curves limiting the lower- and upper-bounds were obtained from parameters of the 25th and 75th percentiles curves, respectively. The 1-pole Cole-Cole parameters are valid for a frequency range from 0.5–20 GHz.

In order to compare the dielectric properties of tumours to other breast tissues, we converted the 1-pole Cole-Cole model to the Debye model. From the reported dielectric property curves, we generated a Debye model fitted to the data points and extracted the Debye parameters for malignant tumours. The fitted parameters are detailed in Table 4.

**Figure 3:**
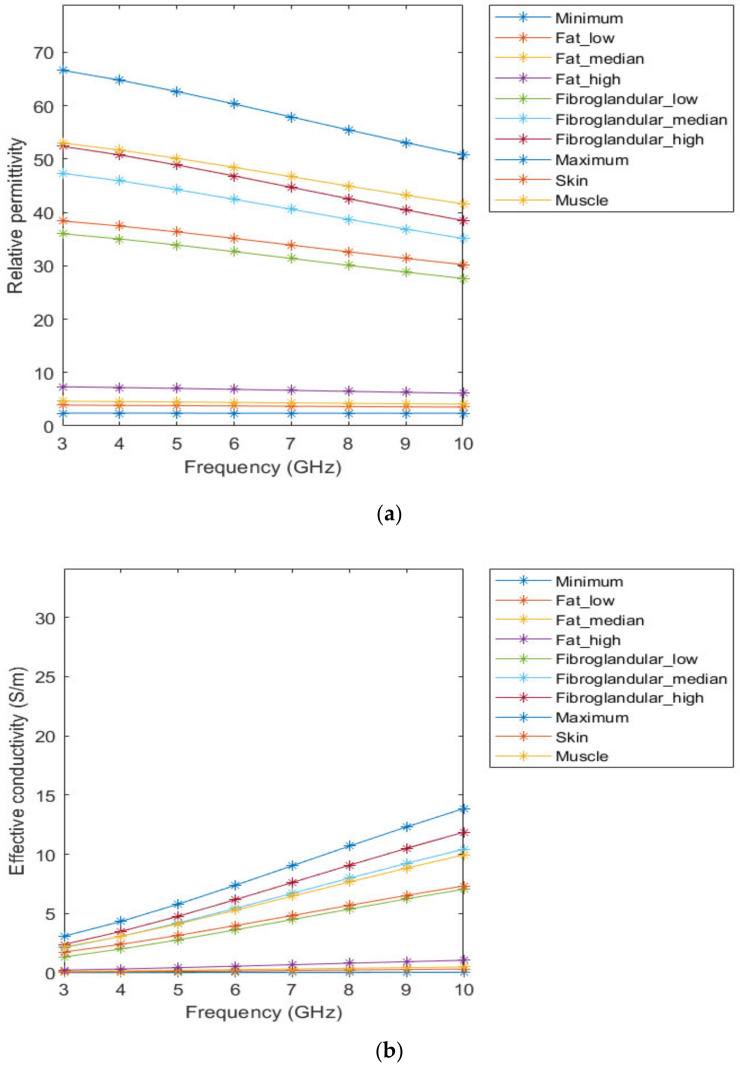
Relative permittivity (**a**) and effective conductivity (**b**) curves of non-tumorous tissues adapted from [64,65] for the frequency range of 3–10GHz.

**Figure 4:**
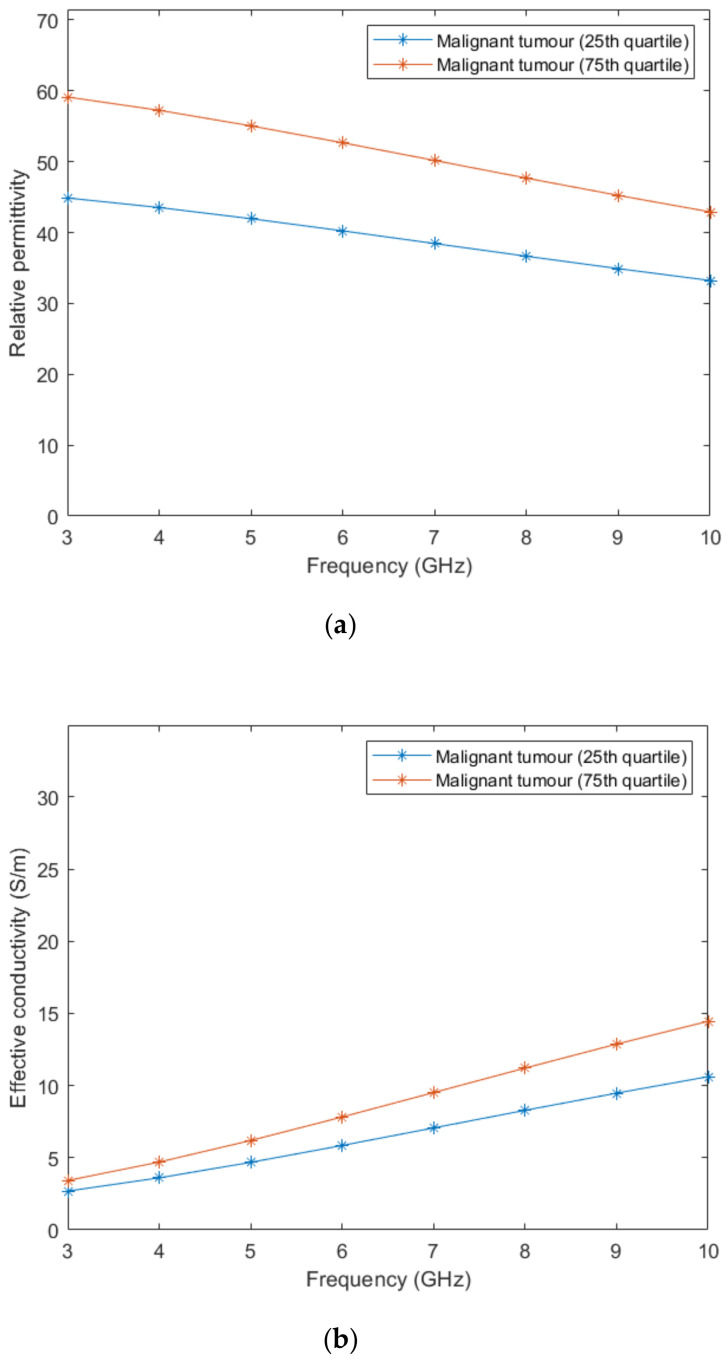
Relative permittivity (**a**) and effective conductivity (**b**) curves obtained from the fitted Debye parameters shown in Table 4.

Figure 3 represents the behaviour of the dielectric properties (relative permittivity and conductivity) as a function of frequency for non-tumorous tissue types. Figure 4 represents the dielectric properties of malignant tumour tissues for different frequencies.

### 2.5. Creation of Breast Region Models

We derived anthropomorphic breast computational models from MRI exams. Such models are the result of the application of the proposed pre-processing and segmentation pipelines, detailed above. These models include skin, fat, fibroglandular, and muscle tissues, as well as benign and malignant tumours, segmented from the MR images. Microwave-frequency properties of breast tissues at 3, 6, and 9 GHz are also available in our models.

## 3. Results

This section shows the results of the developed segmentation pipeline applied to breast MRI exams for breast tissue segmentation and the estimation of the dielectric properties of the segmented tissues from MRI exams. In this paper, we depicted the results of the steps previously described for two exams (one with a benign tumour and the other with an extremely heterogeneous malignant tumour). Both models include the dielectric properties estimated for 6 GHz. Additionally, the repository contains more breast models, including benign and malignant tumours, with the estimated dielectric properties also for 3 GHz and 9 GHz.

### 3.1. Pre-Processing Pipeline

#### 3.1.1. Registration

**Figure 5:**
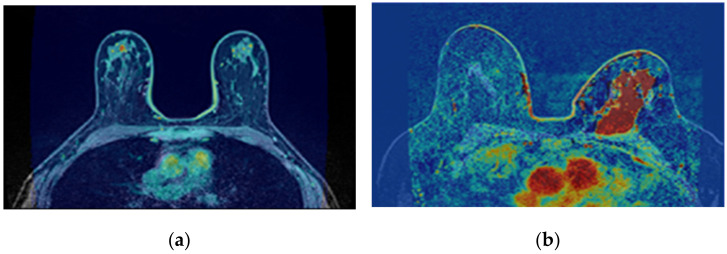
Resulting images from the registration of the transverse SUB-DCE-fl3D image (moving image) to the coronal T1-w Dixon-W image (static image), in a breast exam with a (**a**) benign and (**b**) a malignant tumour.

The transverse SUB-DCE-fl3D sequence used for tumour segmentation was initially registered to the coronal T1-w Dixon sequence. Figure 5 shows the resulting images of the alignment of the two images in T1-w Dixon spatial reference.

#### 3.1.2. Bias Field Correction

**Figure 6:**
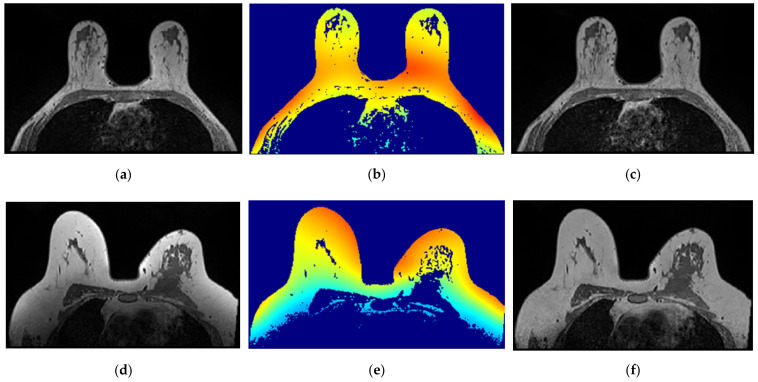
Bias field correction performed on a breast MRI exam with a benign tumour (top) and a malignant tumour (bottom). (**a**,**d**) show one slice from each exam before the application of the bias field correction filter. (**b**,**e**) show the inhomogeneity between the voxel intensities. Red regions represent areas where the inhomogeneity between voxel intensities is larger. (**c**,**f**) show the same slices after correction of the bias field.

The correction of the bias field is a common step to T1-w Dixon-I, T1-w Dixon-W, and T1-w Dixon-F images, as well as SUB-DCE-fl3D images. Figure 6 illustrates (a,d) the original T1-w Dixon-I images, (b,e) the bias field present in each image, and (c,f) the corrected images, for an exam with a benign tumour (top) and a malignant tumour (bottom).

#### 3.1.3. Image Filtering

**Figure 7:**
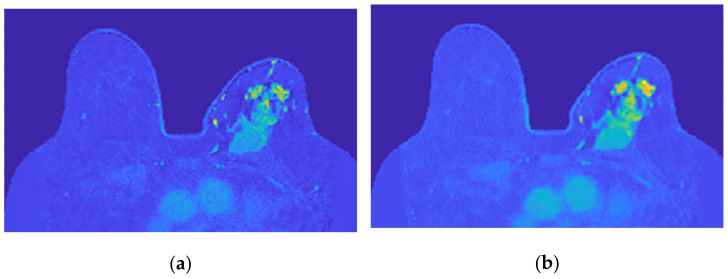
Median filter for edge smoothing: (**a**) image pre-filtering and (**b**) image post-filtering.

The application of a median filter to the pre-processed image, with a tumour larger than 1 cm, smooths the edges, facilitating the breast tumour segmentation process. Figure 7 depicts the application of a median filter for edge smoothing.

### 3.2. Image Segmentation

#### 3.2.1. Breast Region

- Step 1: Fat mask + removal of organs inside the thoracic cavity

**Figure 8:**
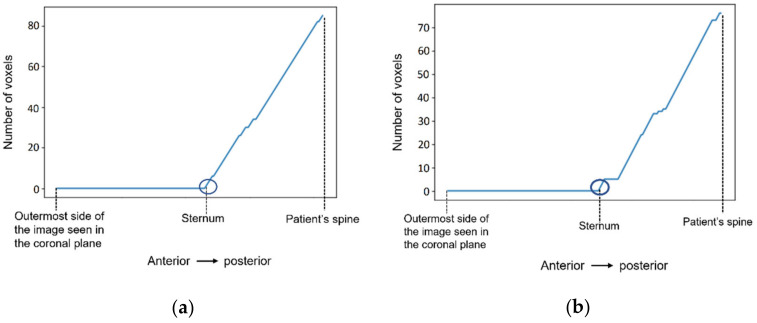
The number of voxels with intensity higher than the mean intensity of the input image (T1-w Dixon-W image) was counted to detect the coordinates of the sternum in (**a**) the exam with the benign tumour, and in (**b**) the exam with the malignant tumour.

Figure 8 shows a representation of the number of voxels with an intensity higher than the mean intensity of the T1-w Dixon-W image counted at the centre of the body, starting outside the patient’s body, and finishing at the patient’s spine. The coordinates of the sternum correspond to the outer-most highlighted voxel. The coordinates of the centre of the body are selected, and then we sweep the coordinate along the coronal plane. The coordinate for the coronal plane that indicates the first sternum voxel is identified in Figure 8 by a dark blue circle.

**Figure 9:**
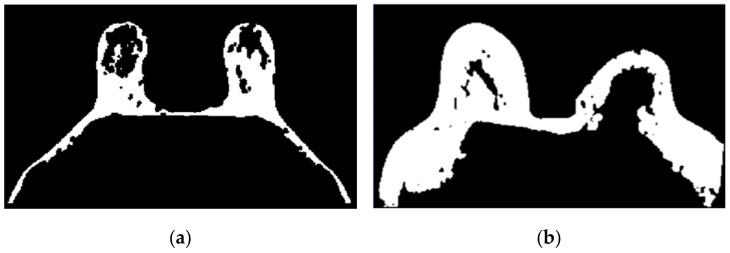
Output of the region growing algorithm applied to (**a**) the exam with the benign tumour and to (**b**) the exam with the malignant tumour.

Figure 9 shows the result of the region growing algorithm applied to the T1-w Dixon-F images. For both exams, the region growing algorithm separates the fat tissue of the breast region from the fat tissue in the thoracic cavity, and the watershed transform from markers was not necessary.

- Step 2: Skin + Fibroglandular + Fat mask

**Figure 10:**
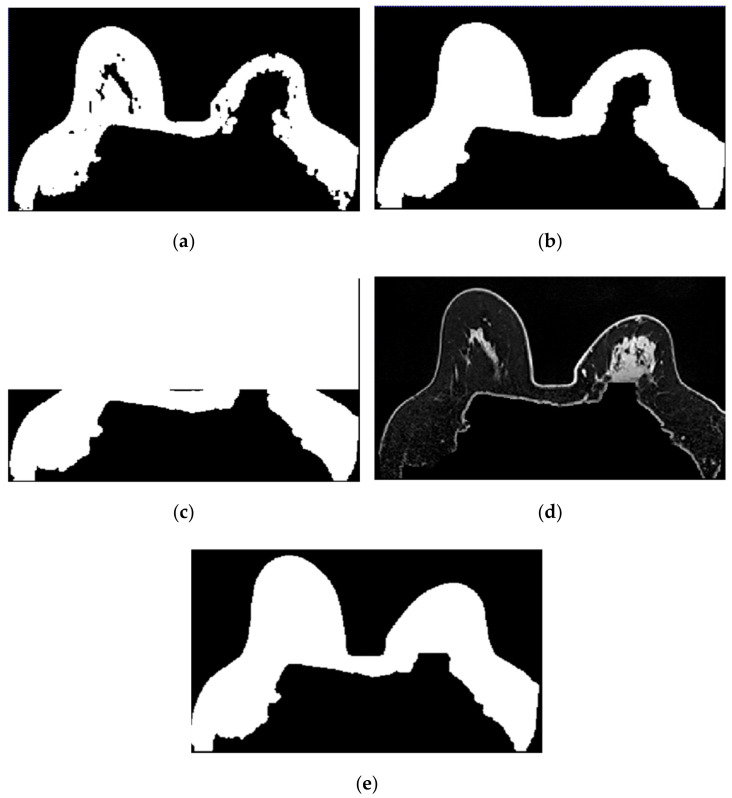
Step-by-step images obtained in Step 2. (**a**) Mask representing the fat tissue obtained from the region growing algorithm. (**b**) Mask obtained from region growing, dilated by a structuring element of radius 3, to include the skin. (**c**) Dilated mask with the anterior region of the body whited out. (**d**) Image obtained by multiplying the image in (**c**) and the original T1-w Dixon-W image. (**e**) Resulting mask after thresholding the image represented in (**d**).

In Figure 10, we depict the several steps followed to obtain a breast mask with fat, skin, and fibroglandular tissues described in Step 2 for the exam with the malignant tumour. Figure 10a represents the fat mask resulting from Step 1. After dilating the fat mask obtained from region growing (Figure 10b) and whiting out the anterior side of the body (Figure 10c), we multiplied the resulting mask and the T1-w Dixon-W image (Figure 10d). After thresholding, we obtain a mask of the breast region (Figure 10e).

**Figure 11:**
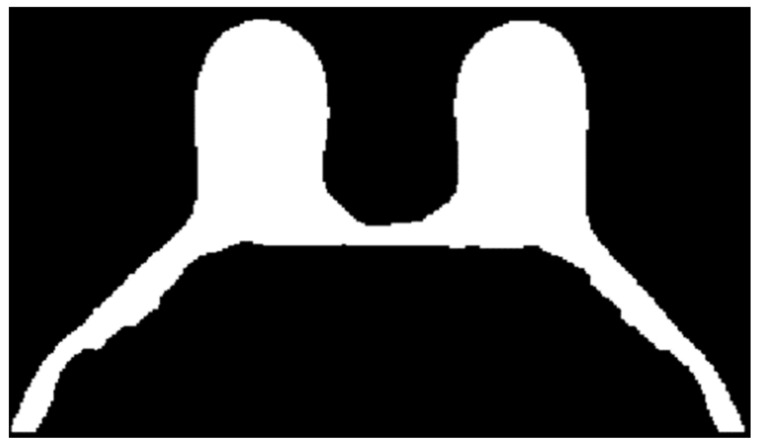
Mask obtained after Step 2 of our methodology for the exam with the benign tumour.

Figure 11 shows the breast mask obtained after concluding Step 2 for the exam with the benign tumour.

- Step 3: Mask evaluation

For the exam with the benign tumour, the mean-squared error value between the original mask and its flipped image, is 4.8%. For the exam with the malignant tumour, the mean-squared error value is 11.1%. As observed in Figure 11, the mask for the exam with the benign tumour is complete, and the mask for the exam with the malignant tumour (Figure 10e) has a hole where the tumour invades the pectoralis muscle.

- Step 4: Mask for an exam with an invasive tumour (optional)

**Figure 12:**
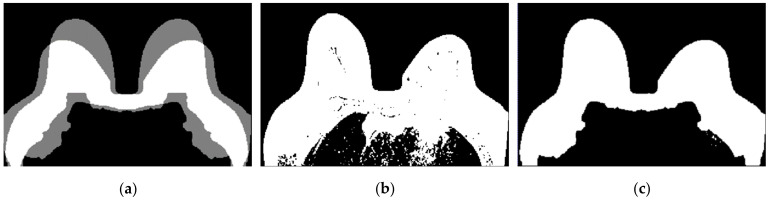
(**a**) Superimposition of the original mask from Step 2 and its flipped image. (**b**) Binarised T1-w Dixon-I image using the mean voxels intensity as threshold. (**c**) Resulting mask of the intersection of (**a**,**b**), after the application of SimpleITK’s BinaryFillholeImageFilter.

To complete the mask for the exam with the malignant tumour, the method described in Step 4 was followed. Figure 12a shows the superimposition of the mask obtained from Step 2 and its flipped image. Figure 12b corresponds to the binarised T1-w Dixon-I image using the mean voxels intensity as threshold. Figure 12c corresponds to the resulting output of Step 4.

- Step 5: Segmentation of skin and breast/chest wall boundary

**Figure 13:**
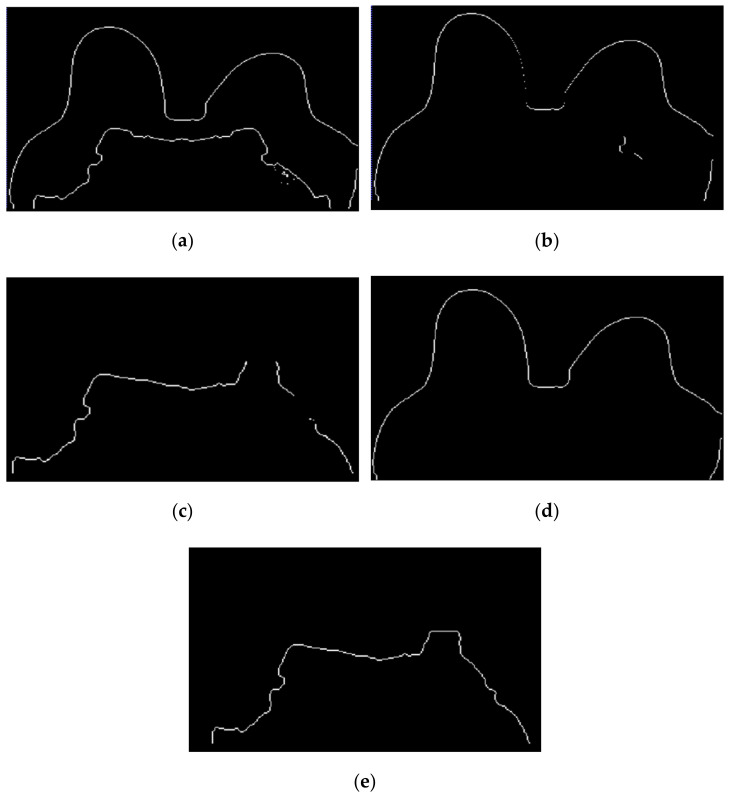
(**a**) Contour of the breast mask. (**b**) Skin contour (1-voxel-thick) after scanning the voxels of the contour breast mask from left to right, top to bottom and right to left. (**c**) Breast/chest wall boundary contour (1-voxel-thick) obtained after subtracting the contour represented in (**b**) from the contour represented in (**a**). Final (**d**) skin and (**e**) breast/chest wall boundary contours obtained after Step 5.

Figure 13 shows, in detail, the steps followed to obtain the skin and breast/chest wall boundaries for the exam with the malignant tumour. After scanning the voxels in the breast contour (Figure 13a), we obtained a 1-voxel-thick skin contour (Figure 13b). An artefact corresponding to the breast/chest wall boundary appears in Figure 13b since part of the skin in the original T1-w Dixon-W image was not visible. The 1-voxel-thick chest wall boundary contour is shown in Figure 13c. The final skin contour, represented in Figure 13d, corresponds to the largest connected component of the subtraction between Figure 13a,c. The final contour of the breast/chest wall boundary, in Figure 13e, corresponds to the subtraction between Figure 13a,d.

**Figure 14:**
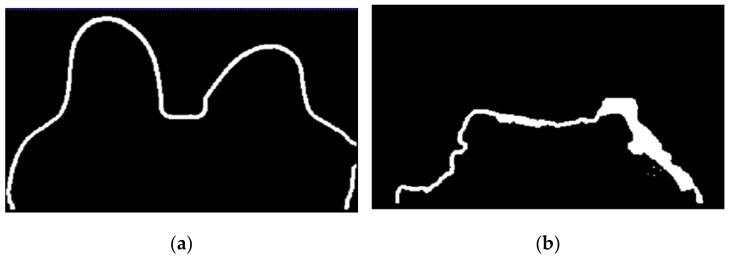
(**a**) Skin contour obtained for the exam with the malignant tumour, and corresponding (**b**) breast/chest wall boundary obtained after following Step 5.

The skin and breast/chest wall boundaries obtained after following Step 5 are presented in Figure 14, for the exam with the malignant tumour.

**Figure 15:**
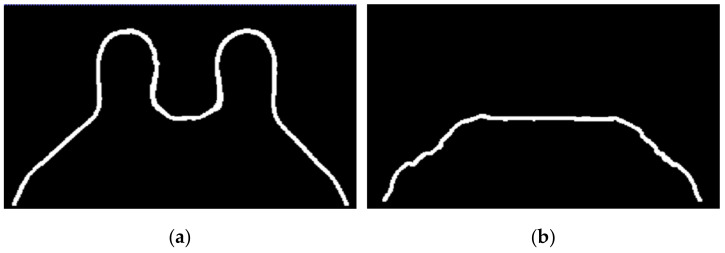
(**a**) Skin contour for the exam with the benign tumour, and the corresponding (**b**) breast/chest wall boundary obtained after following Step 5.

In Figure 15, the skin and breast/chest wall boundaries obtained after following Step 5 are presented for the exam with the benign tumour.

- Step 6: Skin evaluation

**Figure 16:**
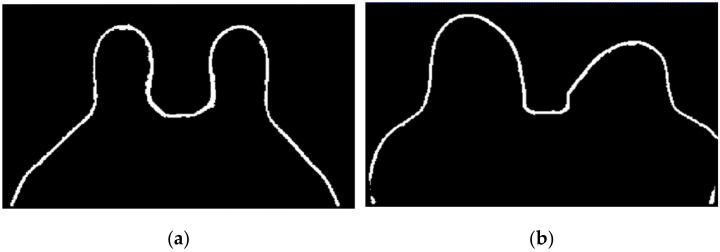
Skin mask obtained after step 6 for (**a**) the exam with the benign tumour, and for (**b**) the exam with the malignant tumour.

Figure 16 shows the output of Step 6 for the exams with the benign and the malignant tumours.

**Figure 17:**
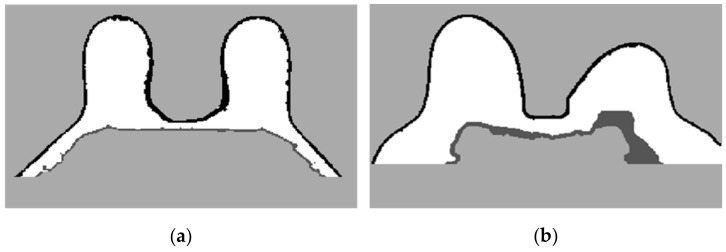
Label map obtained from the processing pipeline. Background is labelled as 0, foreground, which includes fat and fibroglandular tissues, are labelled as 1, the skin is labelled as −2 and the breast/chest wall boundary is labelled as −1. Label maps for (**a**) the exam with the benign tumour, and for (**b**) the exam with the malignant tumour.

Figure 17 shows the final label map obtained from the segmentation pipeline, as previously described. The labels for each component are as follows: 0 for the background, 1 for the foreground (including fat and fibroglandular tissues), −2 for the skin and −1 for the chest/breast wall boundary.

#### 3.2.2. Tumour Segmentation

**Figure 18:**
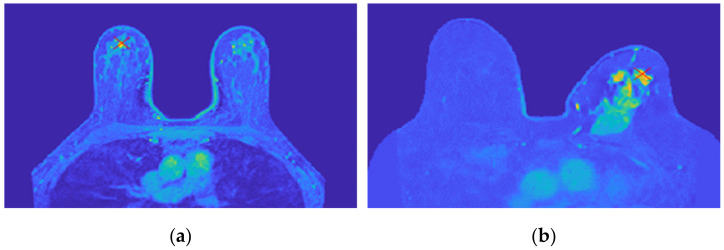
Location of the 3D region growing algorithm seed marked with a red cross in (**a**) the exam with the benign tumour and (**b**) the exam with the malignant tumour.

Figure 18 shows the location of the seed marked with a red cross for (a) the benign tumour and (b) the malignant tumour, which is the starting point of the 3D region growing algorithms. The choice of the seed was automatic for (a) and manual for (b).

**Figure 19:**
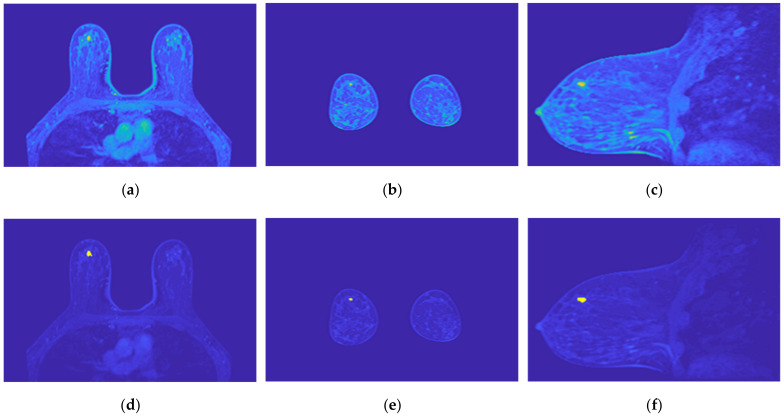
Output of the region growing algorithm for the exam with the benign tumour: (**a**–**c**) correspond to transverse, coronal, and sagittal planes of the pre-processed SUB-DCE-fl3D image for tumour segmentation, respectively; (**d**–**f**), illustrate the superimposition of the tumour mask (yellow) to the pre-processed SUB-DCE-fl3D image in the transverse, coronal, and sagittal planes.

Figure 19 illustrates the output of the region growing algorithm for the exam with the benign tumour, in the three anatomical planes.

**Figure 20:**
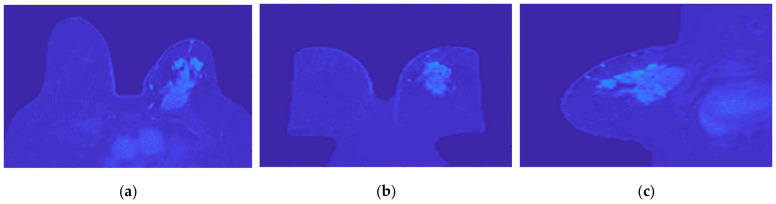

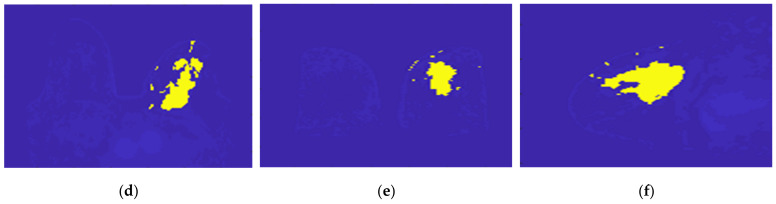
Output of the region growing algorithm for the exam with the malignant tumour: (**a**–**c**) correspond to transverse, coronal, and sagittal planes of the pre-processed SUB-DCE-fl3D image for tumour segmentation, respectively; (**d**–**f**) illustrate the superimposition of the tumour mask (yellow) to the pre-processed SUB-DCE-fl3D image in the transverse, coronal, and sagittal planes.

Figure 20 illustrates the output of the region growing algorithm for the exam with the malignant tumour, in the three anatomical planes.

**Figure 21:**
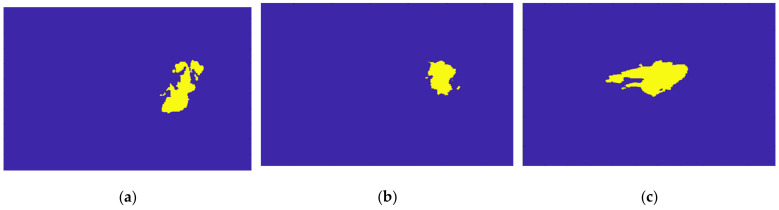
Output of the Hoshen-Kopelman algorithm plus manual correction of the tumour mask: (**a**–**c**)correspond to transverse, coronal, and sagittal planes.

Figure 21 illustrates the output of the Hoshen-Kopelman algorithm plus manual correction of the tumour mask, in the three anatomical planes. Hoshen-Kopelman isolates tumours by removing regions that do not belong to the tumour tissue. However, for extremely heterogenous cases such as the one presented in this paper, the Hoshen-Kopelman algorithm was not enough to isolate the tumour since nearby blood vessels are present in contiguous regions; therefore, manual correction was required to obtain a binary mask of the tumour.

**Figure 22:**
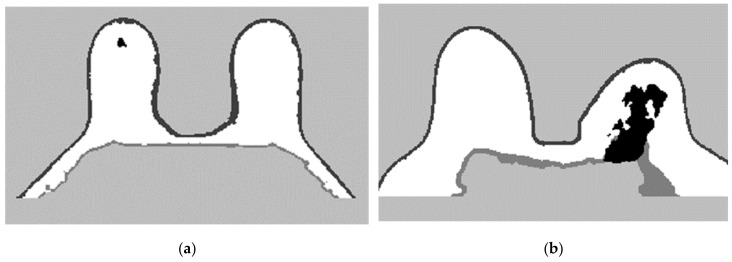
Final label map for (**a**) the exam with the benign tumour and for (**b**) the exam with the malignant tumour.

The final label maps, including the tumour segmentation are shown in Figure 22. Tumour label corresponds to −3.

### 3.3. Dielectric Properties

**Figure 23:**
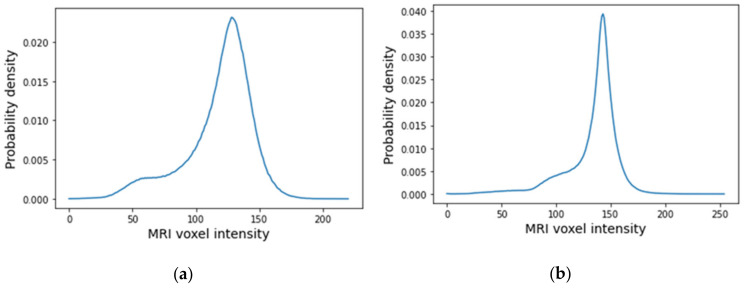
Histogram of MRI voxel intensities for (**a**) the exam with the benign tumour and for (**b**) the exam with the malignant tumour.

The histograms of the breast data of the fat and fibroglandular tissues (labelled as 1 in the label maps) for both exams are represented in Figure 23. For the benign tumour, it is possible to separate fat and fibroglandular tissues through the histogram (Figure 23a) using parameters described in Table 1. The voxel intensities corresponding to fat and fibroglandular tissues can be separated in the histogram of the exam with the malignant tumour (Figure 23b) using parameters described in Table 2. Table 5 includes a list of the parameters extracted from the Gaussian Mixture Model used to obtain the piecewise-linear mapping curve between the MRI voxel intensity and the dielectric properties.

**Figure 24:**
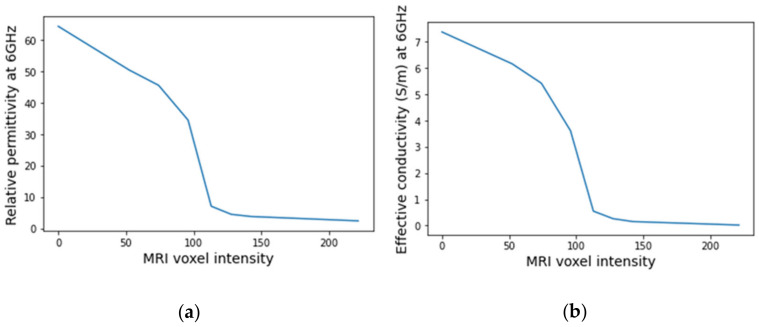
Example of the piecewise linear mapping obtained at 6 GHz (**a**) of the relative permittivity and (**b**) effective conductivity for the exam with the benign tumour.

An example of a piecewise-linear map between the relative permittivity/effective conductivity at the frequency 6 GHz and the MRI voxel intensity for the exam with the benign tumour is represented in Figure 24.

**Figure 25:**
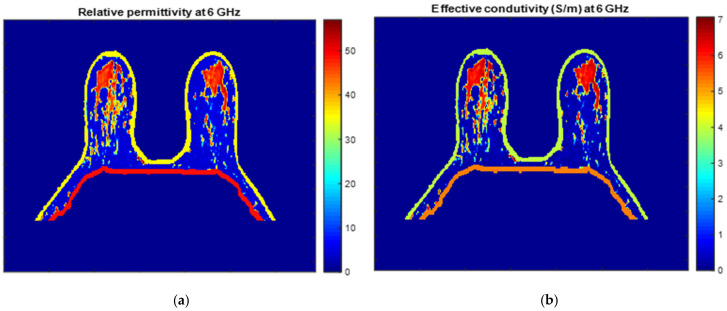
Map of the dielectric properties for the exam with the benign tumour: (**a**) relative permittivity at 6 GHz and (**b**) effective conductivity (S/m) at 6 GHz.

**Figure 26:**
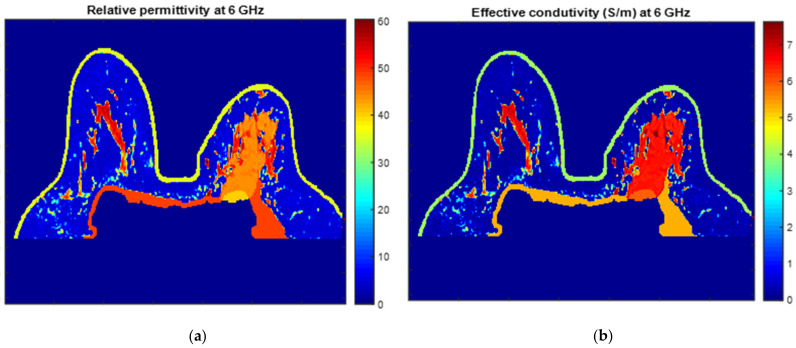
Map of the dielectric properties for the exam with the malignant tumour: (**a**) relative permittivity at 6 GHz and (**b**) effective conductivity (S/m) at 6 GHz.

Figure 25 and Figure 26 show the dielectric property maps for the relative permittivity and effective conductivity (S/m) at 6 GHz for the exams with the benign and malignant tumours, respectively. We would like to note the different colour scales in both figures; for the benign tumour, the relative permittivity and conductivity values vary between 0 and 60.3, and 0 and 7.37 (S/m), respectively; for the malignant tumour, the relative permittivity and conductivity values vary between 0 and 60.3, and 0 and 7.82 (S/m), respectively.

### 3.4. Breast Region Models Repository

The repository is available for download in GitHub (https://github.com/acpelicano/breast_models_repository, created on 27 October 2021). Initially, it only included two breast models, as portrayed in this paper: one of a benign infra-centimetric tumour of rounded shape; and the other of a malignant invasive tumour of irregular shape, with approximately 8 cm along its major axis, and extremely heterogenous regarding voxels intensities. The models include the dielectric properties of the breast tissues estimated at frequencies of 3, 6, and 9 GHz. Models from the full dataset collected from patients, as described in Section 2.1, will be added as the processing of each is completed.

## 4. Discussion and Conclusions

We presented an image processing pipeline to segment healthy breast tissues and tumours inspired by the available literature. Most models for the breast region are based on clustering algorithms or the identification of air/breast and breast/chest wall boundaries. However, the fat tissue inside the thoracic cavity, with no clear separation from other tissues inside the breast, hinders the correct identification of the breast region. Additionally, the identification of the breast/chest wall boundary is especially difficult in the presence of a heterogeneous malignant tumour that is invading the pectoralis muscle, as is the case presented in this paper.

The proposed segmentation pipeline can separate the fat tissues in the breast and inside the thoracic cavity by applying the region growing algorithm. In addition, we suggested using the watershed transform from markers when the region growing algorithm fails to provide a good separation between both fat tissues. If there is no prior knowledge of whether an invasive tumour is present in the MRI image, and to account for the area where it invades the pectoralis muscle, we proposed a method that automatically evaluates the breast mask after region growing/watershed transform and fills that area.

We developed a semi-automatic segmentation pipeline particularly robust when segmenting breast tumours with a varying level of heterogeneity regarding voxel intensity. The proposed pipeline is flexible since it does not operate under the assumption that, generally, biological tissues are well separated within a grayscale image. For the tumour segmentation pipeline, we used a 3D region growing algorithm. Although not fully automatic, it suggests using the highest intensity point in a slice containing the tumour as the seed point and using, as the lower threshold, the mean minus three times the standard deviation of all body voxels intensity values. This methodology provided better results when compared to widely used segmentation methods, such as K-means. The Hoshen-Kopelman algorithm eliminated voxels included in the growing region of the tumour that did not belong to that tissue. For the malignant tumour presented in this paper, we also performed manual correction of the tumour mask.

We estimated the dielectric properties for the malignant tumour tissue from the reported Debye parameters. For the benign tumour tissue, literature reports that the dielectric properties of these tissues are similar to the properties of lower-adipose-content normal breast tissues; hence, we estimated the dielectric properties using interpolation between the curves of healthy breast tissues.

In this paper, we presented two realistic breast models where skin, muscle, fat, fibroglandular, and tumour tissues (malignant and benign) are identified. In addition, the models in our paper include the dielectric properties calculated for the frequency of 6 GHz. Anatomically realistic breast and tumour (benign and malignant) models, portraying the realistic shapes of these tissues, are available to all with estimated dielectric properties for frequencies of 3, 6, and 9 GHz in a public repository.

## Figures and Tables

**Table 1:** Linear piecewise mapping between the voxel intensities and the corresponding dielectric property curve for the histogram of an exam where the contribution of the gaussian curves of fat and fibroglandular tissue are separated.

Dielectric Property Curves	Voxel Intensity
Minimum	0
Fibroglandular_low	$\mu_{fg}-\sigma_{fg}$
Fibroglandular_median	$\mu_{fg}$
Fibroglandular_high	$\mu_{fg}+\sigma_{fg}$
Fat_low	$\mu_{fat}-\sigma_{fat}$
Fat_median	$\mu_{fat}$
Fat_high	$\mu_{fat}+\sigma_{fat}$
Maximum	Maximum intensity of the image

**Table 2:** Linear piecewise mapping between the voxel intensities and the corresponding dielectric property curve for a histogram of an exam where the fat tissue is predominant over the fibroglandular tissue and the contributions of the gaussian curves of fat and fibroglandular tissues are not clearly separated.

Dielectric Property Curves	Voxel Intensity
Minimum	0
Fibroglandular_low	$2\times \mu_{fg}-M_{fg}$
Fibroglandular_median	$\mu_{fg}$
Fibroglandular_high	$M_{fg}=\mu_{fat}-\sigma_{fat}-\delta$
Fat_low	$\mu_{fat}-\sigma_{fat}$
Fat_median	$\mu_{fat}$
Fat_high	$\mu_{fat}+\sigma_{fat}$
Maximum	Maximum intensity of the image

**Table 3:** Single-pole Debye parameters for the dielectric property curves for each tissue type [64].

	$\varepsilon_{\infty }$	Δε	$\tau \left(ps\right)$	$\sigma_{s}\left(S/m\right)$
Minimum	2.309	0.092	13.00	0.005
Fibroglandular_low	12.99	24.40	13.00	0.397
Fibroglandular_median	13.81	35.55	13.00	0.738
Fibroglandular_high	14.20	40.49	13.00	0.824
Fat_low	2.848	1.104	13.00	0.005
Fat_median	3.116	1.592	13.00	0.050
Fat_high	3.987	3.545	13.00	0.080
Maximum	23.20	46.05	13.00	1.306
Skin	15.93	23.83	13.00	0.831
Muscle	21.66	33.24	13.00	0.886

**Table 4:** Fitted Debye parameters for the dielectric property curves of malignant breast tumours.

**Percentile**	** $\varepsilon_{\infty }$ **	Δε	$\tau \left(ps\right)$	$\sigma_{s}\left(S/m\right)$
25th	12.9	33.9	13.0	1.38
75th	14.6	47.2	13.0	1.60

**Table 5:** Parameters of the Gaussian Mixture Model for the exam with the benign tumour and the exam with the malignant tumour (rounded to the nearest unit).

	Exam with the Benign Tumour		Exam with the Malignant Tumour	
	Voxel Intensity Equations	Voxel Intensity	Voxel Intensity Equations	Voxel Intensity
Minimum	0	0	0	0
Fibroglandular_low	$\mu_{fg}-\sigma_{fg}$	55	$2\times \mu_{fg}-M_{fg}$	104
Fibroglandular_median	$\mu_{fg}$	80	$\mu_{fg}$	115
Fibroglandular_high	$\mu_{fg}+\sigma_{fg}$	104	$M_{fg}=\mu_{fat}-\sigma_{fat}-\delta$	127
Fat_low	$\mu_{fat}-\sigma_{fat}$	113	$\mu_{fat}-\delta_{fat}$	134
Fat_median	$\mu_{fat}$	129	$\mu_{fat}$	143
Fat_high	$\mu_{fat}+\sigma_{fat}$	144	$\mu_{fat}+\delta_{fat}$	152
Maximum	Maximum intensity of the image	221	Maximum intensity of the image	255

## Data Availability

Anatomically realistic breast and tumour (benign and malignant) models with the estimated dielectric properties at frequencies of 3, 6 and 9 GHz are available online at https://github.com/acpelicano/breast_models_repository (created on 27 October 2021).
